# In-Situ Measurement of High-Temperature Proton Exchange Membrane Fuel Cell Stack Using Flexible Five-in-One Micro-Sensor

**DOI:** 10.3390/s16101731

**Published:** 2016-10-18

**Authors:** Chi-Yuan Lee, Fang-Bor Weng, Yzu-Wei Kuo, Chao-Hsuan Tsai, Yen-Ting Cheng, Chih-Kai Cheng, Jyun-Ting Lin

**Affiliations:** Department of Mechanical Engineering, Yuan Ze Fuel Cell Center, Yuan Ze University, Taoyuan 320, Taiwan; fangbor@saturn.yzu.edu.tw (F.-B.W.); alkuo@apfct.com.tw (Y.-W.K.); s1045017@mail.yzu.edu.tw (C.-H.T.); s1005071@mail.yzu.edu.tw (Y.-T.C.); cheng.akai@apfct.com.tw (C.-K.C.); s1020908@mail.yzu.edu.tw (J.-T.L.)

**Keywords:** five-in-one micro-sensor, HT-PEMFC stack, in-situ measurement

## Abstract

In the chemical reaction that proceeds in a high-temperature proton exchange membrane fuel cell stack (HT-PEMFC stack), the internal local temperature, voltage, pressure, flow and current nonuniformity may cause poor membrane material durability and nonuniform fuel distribution, thus influencing the performance and lifetime of the fuel cell stack. In this paper micro-electro-mechanical systems (MEMS) are utilized to develop a high-temperature electrochemical environment-resistant five-in-one micro-sensor embedded in the cathode channel plate of an HT-PEMFC stack, and materials and process parameters are appropriately selected to protect the micro-sensor against failure or destruction during long-term operation. In-situ measurement of the local temperature, voltage, pressure, flow and current distributions in the HT-PEMFC stack is carried out. This integrated micro-sensor has five functions, and is favorably characterized by small size, good acid resistance and temperature resistance, quick response, real-time measurement, and the goal is being able to be put in any place for measurement without affecting the performance of the battery.

## 1. Introduction

Facing the challenges of global climate change and the need to reduce greenhouse gas emissions, people are becoming increasingly aware of the importance of sustainable development and green technology. Therefore, various countries are promoting the green energy industry. In Taiwan, the Executive Yuan launched the “Dawning Green Energy Industry Program” to develop the green energy sector. This program is expected to facilitate research and development into hydrogen energy and fuel cell technology and product development [1]. 

Tan [2] suggested that the unreasonable consumption of energy may inhibit the sustainable development of the national economy. Newly developed energy generation technology must be efficient, clean and safe. Therefore, the fuel cell has become an important new source of energy. 

The fuel cell stack is essential to the commercialization of fuel cells. A good fuel cell stack must exhibit close to ideal flow, reacting gas, temperature distribution and uniform stacking pressure [3]. 

The HT-PEMFC stack is characterized by its portability, high energy conversion efficiency, lack of electrolyte loss, ease of assembly and production, and long operating lifetime [4]. In the last decade, research teams around the world have paid close attention to the high-temperature proton exchange membrane fuel cell stack (HT-PEMFC) stack (120~200 °C) [5,6,7]. 

Harms [8] showed that the voltage of the fuel cell stack is governed by the cathode, particularly the voltage drop at low relative humidity. A high current load can dry the proton exchange membrane and degrade the performance. Therefore, the measurement of both current and voltage is very important. Our research team has successfully embedded micro temperature and voltage sensors inside the HT-PEMFC stack [9,10,11].

The shortcomings of the low-temperature fuel cell stack include the following; (1) the anode catalyst has poor resistance to CO poisoning at low temperature; (2) the perfluorosulfonic acid membrane has good ionic conductivity only at high humidity; (3) the cathodic reduction overpotential is high; (4) liquid water and heat removal management [12] delays bulk production. However, problems of the HT-PEMFC stack, such as durability of the membrane material, catalyst corrosion, local flow, pressure, temperature, and voltage and current nonuniformity inside the fuel cell stack, must be solved before such a fuel cell can be commercialized. This investigation concerns the in-situ monitoring of local flow, pressure, temperature, voltage and current in the HT-PEMFC stack.

## 2. Sensing Principle and Design of Five-in-One Micro-Sensor

### 2.1. Sensing Principle of Five-in-One Micro-Sensor

#### 2.1.1. Micro Temperature Sensor 

**Figure 1:**
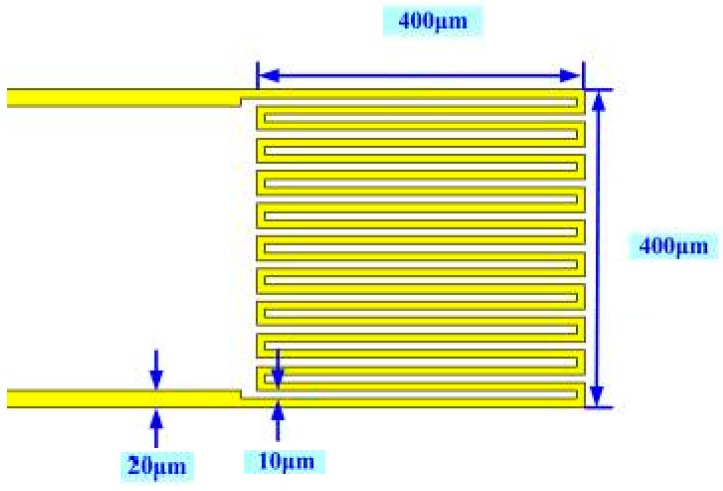
Design of micro temperature sensor.

Figure 1 displays the design of a micro temperature sensor. The sensing principle is that, since gold has a positive temperature coefficient (PTC), as the ambient temperature rises, its resistivity increases, as according to Equation (1):

(1) $$\alpha =\frac{1}{\rho_{0}}\frac{d\rho }{dT}$$

When a resistance-based temperature detector is used in the range of linear variation of resistance with temperature, Equation (1) reduces to Equation (2).

(2) $$R_{t}=R_{r}(1+\alpha_{1}\Delta T)$$

Equation (2) can be changed to Equation (3).

(3) $$\alpha_{1}=\frac{R_{t}-R_{r}}{R_{r}(\Delta T)}$$

#### 2.1.2. Micro Voltage Sensor 

**Figure 2:**
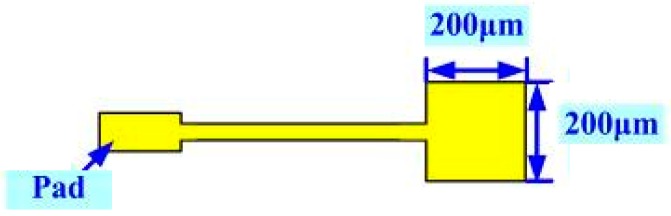
Design of micro voltage sensor.

Figure 2 displays the design of the micro voltage sensor. A 200 μm × 200 μm sensing area of the micro voltage sensor is exposed. The conductors are insulated by an insulating layer, ensuring that the voltage that is detected herein by the flaky probe deep in the high-temperature fuel cell stack is detected locally. The voltage is measured between the probe (inserted in the cathode flow-field) and the anode plate.

#### 2.1.3. Micro Pressure Sensor 

**Figure 3:**
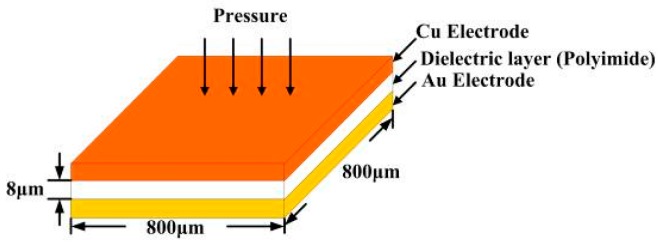
Design of capacitive micro pressure sensor.

The capacitive sensing area is designed as 800 μm × 800 μm, as presented in Figure 3. 

#### 2.1.4. Micro Flow Sensor

**Figure 4:**
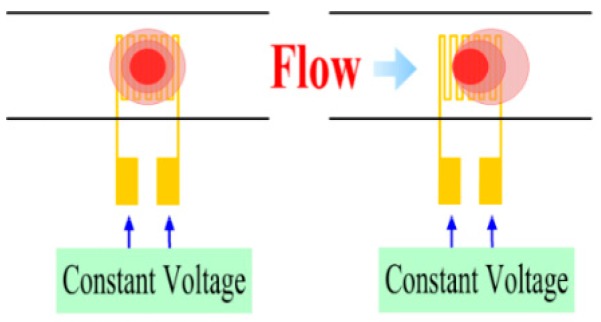
Sensing principle of micro flow sensor.

Figure 4 shows the sensing principle of a hot-wire micro flow sensor. Figure 1 also displays the design of a micro flow sensor.

#### 2.1.5. Micro Current Sensor 

**Figure 5:**
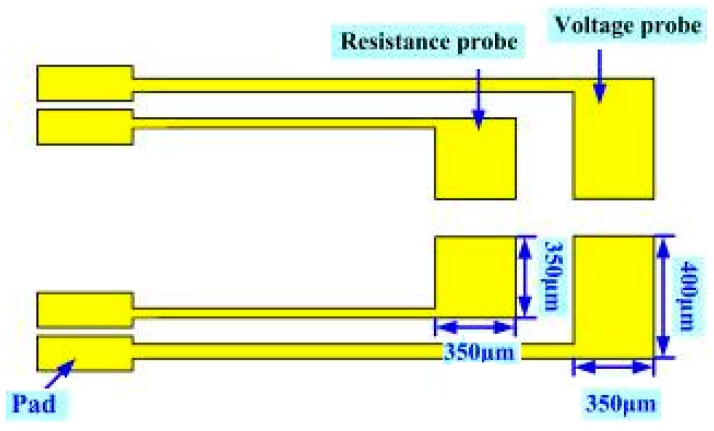
Design of micro current sensor.

Micro current sensors are designed herein with sizes 350 μm × 350 μm and 400 μm × 350 μm integrated in the thickness of a 40 μm stainless steel sheet (stainless steel foil) of the flexible substrate, as displayed in Figure 5. As in a micro voltage sensor, the front-end probe is exposed while the other parts are covered with an insulating layer, ensuring that the probe penetrating into the fuel cell stack can detect the current of the local specific area, and the external measuring instrument measures the voltage difference and the resistivity.

#### 2.1.6. Finished Product of Five-in-One Micro-Sensor 

**Figure 6:**
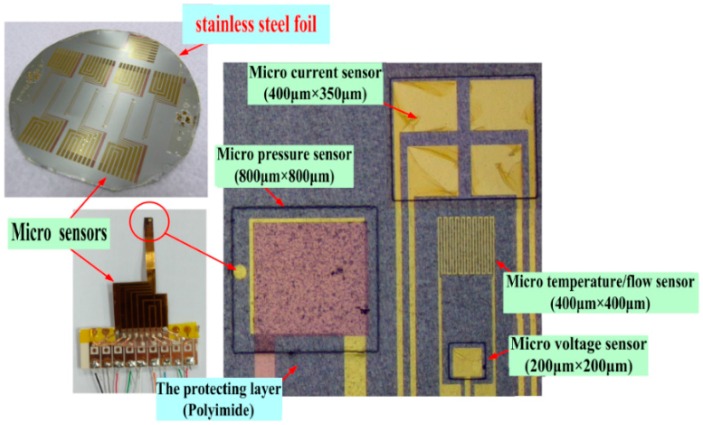
Real product and optical micrograph of five-in-one micro-sensor.

Figure 6 shows the real product and optical micrograph of the five-in-one micro-sensor. A 4 in stainless steel substrate can be made into seven five-in-one micro-sensors. The five-in-one micro-sensor has many important characteristics—compactness, resistance against acid corrosion, favorable thermal tolerance, a short response time, and the ability to make measurements in real time.

#### 2.1.7. Sensor Calibration

When the five-in-one micro-sensor is inserted into the HT-PEMFC stack to make local measurements in real time, corrections must be made for its temperature, the flow rate and the pressure, to yield electrical measurements that correspond to physical quantities. After the device has been calibrated three times, the mean value is obtained and the reliability of its signals is tested.

**Figure 7:**
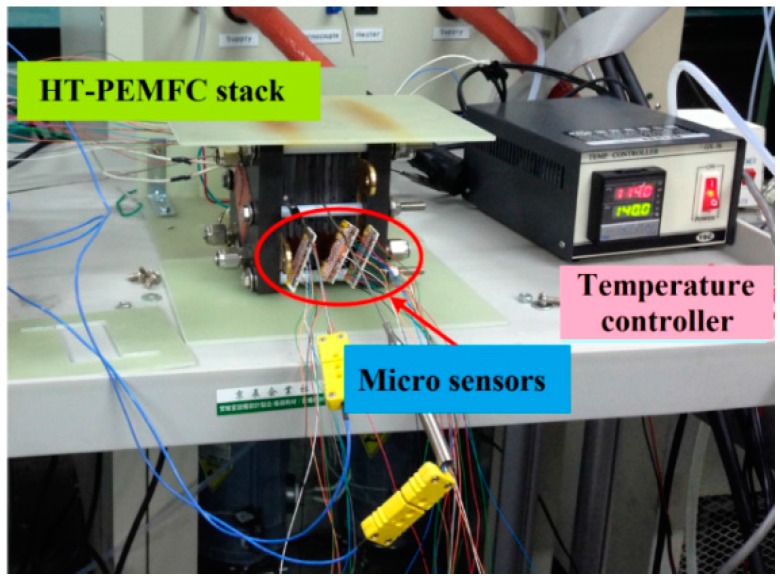
HT-PEMFC stack as the temperature calibration benchmark to calibrate the temperature of the five-in-one micro-sensors.

In this work, the external temperature controller of the HT-PEMFC stack is used directly to provide the benchmark to calibrate the temperature of the five-in-one micro-sensors, as presented in Figure 7. The calibration curves of the micro temperature sensors that are embedded inside the HT-PEMFC stack are obtained from three calibrations at temperatures from 140 °C to 190 °C. The micro temperature sensor has an accuracy better than 0.5 °C. 

Regarding the calibration based on pressure, a Wayne Kerr Electronics (London, UK) 4230 LCR (Inductance, Capacitance, Resistance) meter was used to capture the relevant data. The range of the capacitances that can be measured using the instrument is 0.01 pF~1 F, and its precision is ±0.1%. The micro pressure sensor has an accuracy better than 0.01 kgf/cm^2^.

The micro flow rate sensors can be calibrated in a stable temperature field in air which the power supply generates at the given voltage. The range of flow rates in the calibration is 0~30 L/min. The micro flow rate sensor has an accuracy of better than 0.1 L/min.

## 3. Test Result of Constant Current (5, 13, 20 A) Output

**Figure 8:**
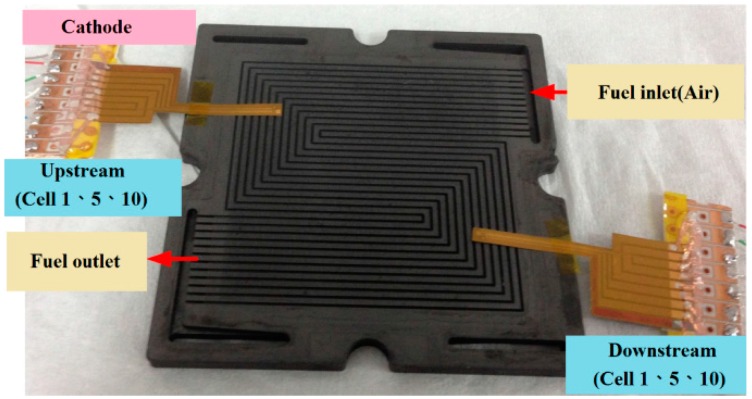
Position of five-in-one micro-sensors in cathode channel plates.

An experiment is carried out on the HT-PEMFC stack at a stable temperature and an open circuit voltage (OCV). In this experiment, the operating temperature of the fuel cell stack is 160 °C, as indicated in Table 1; the size of fuel cell stack is 33 cm^2^ and the reaction area is 31.4 cm^2^. The layers are made of graphite because it resists corrosion. Five-in-one micro-sensors are installed upstream and downstream in Cells 1, 5 and 10, as displayed in Figure 8. The machine gives the anode and cathode fixed, not-humidified gases (anode H_2_: 5 slpm; cathode Air: 30 slpm), and different loads are given a constant current (5, 13, 20 A); the output time is 60 min. The local temperature, voltage, pressure, flow rate and current in the HT-PEMFC stack are obtained by real-time continuous measurement using an NI PXI 2575 data acquisition unit. The embedded five-in-one micro-sensors inside the HT-PEMFC stack cover the reaction area of the MEA (Membrane Electrode Assembly) to prevent the gas from reacting with the covered MEA (the HT-MEA used in this study is a commercially available Advent Energy Company’s (Patras, Greece) HT-MEA; the specifications are in Table 2). However, since the five-in-one micro-sensors are very small, their total area equals only 1.34% of the MEA reaction area. The embedded five-in-one micro-sensors in the HT-PEMFC stack alter the performance of the stack by around 1.3%.

### 3.1. Local Temperature and Voltage Distributions in Different Cells 

The external voltages of different cells and the local temperature and voltage inside the HT-PEMFC stack, measured between the probe (inserted in the cathode flow-field) and the anode plate at an operating temperature of 160 °C, are discussed and analyzed as follows.

**Figure 9:**
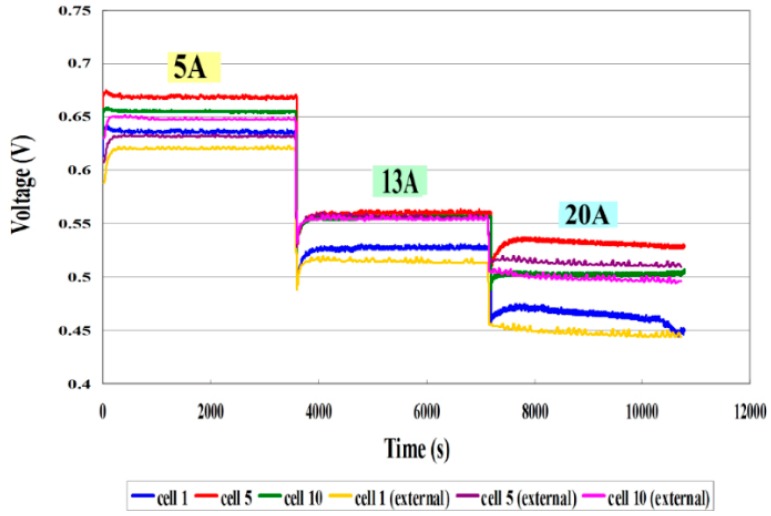
Comparison of internal and external voltages.

**Figure 10:**
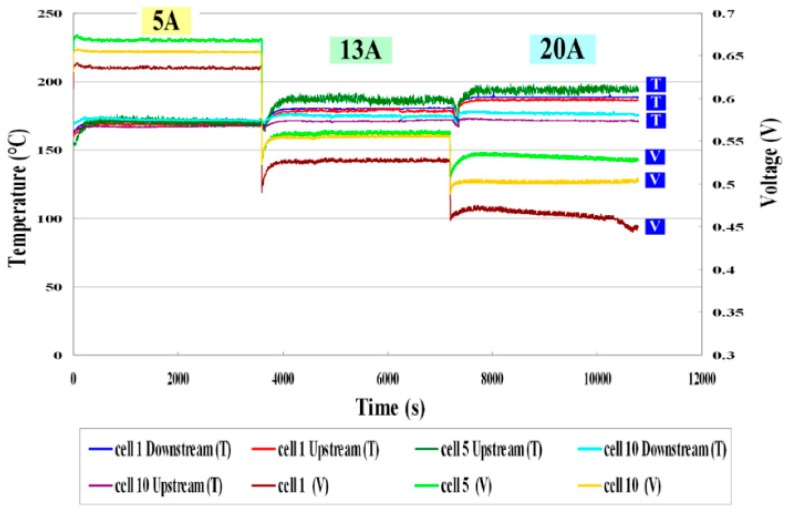
Comparison of local temperatures and voltages in different cells.

1. Figure 9 compares the internal and external voltages. The voltage curve is smooth at a constant current (5, 13 A), because the electrochemical reaction is uniform. At a high current (20 A), the internal electrochemical reaction is violent, and the voltage distribution is nonuniform. The internal local voltage is measured using a micro voltage sensor. The curve of the variation of the internal voltage is consistent with that of the external voltage.
2. Figure 10 plots the local temperature and voltage in different cells. The temperature increases gradually with the current (5, 13, 20 A), because a higher operating current causes more heat to be released from the electrochemical reaction, and as the current increases to a high value (20 A), the thermal nonuniformity becomes gradually worse. Cell 5 has the highest temperature, perhaps because Cell 5 is located in the center of the HT-PEMFC stack. The gases on both ends transfer heat from the front end of the stack to the tail end. The internal electrochemical reaction is vigorous at a high current (20 A), and heat is concentrated, causing Cell 5 to be the hottest.

### 3.2. Local Pressure Distribution in Different Cells 

**Figure 11:**
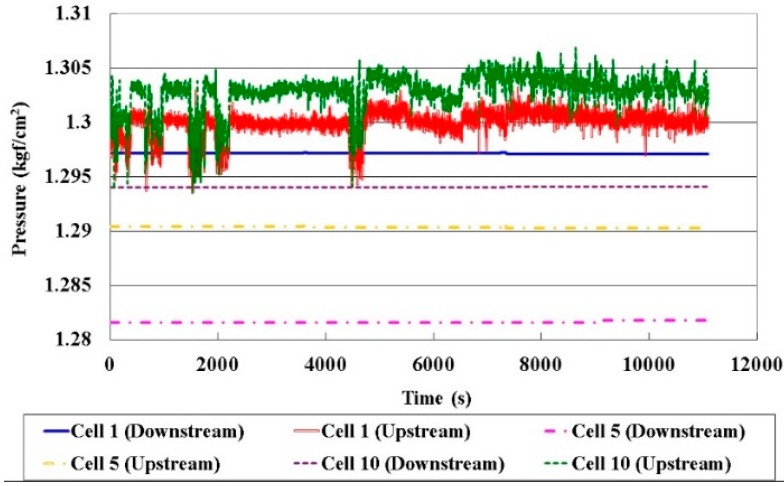
Comparison of local pressures in different cells.

Figure 11 plots the local pressure in different cells, and the pressure control in the experimental work is 1.3 kgf/cm^2^. The local pressure in the HT-PEMFC stack does not vary as the current (5, 13, 20 A) increases. The upstream pressure slightly exceeds the downstream pressure. The mean pressure in the HT-PEMFC stack is 1.291 kgf/cm^2^. The upstream end pressure of Cell 1 and Cell 10 is unstable, but is stabilized at the downstream end. The difference between the end pressure upstream and that downstream in Cell 5 is small, so the pressure in the HT-PEMFC stack is stable. This can be used to assess whether the design of the flow field inside the cell stack is good or not.

### 3.3. Local Flow Distribution in Different Cells

**Figure 12:**
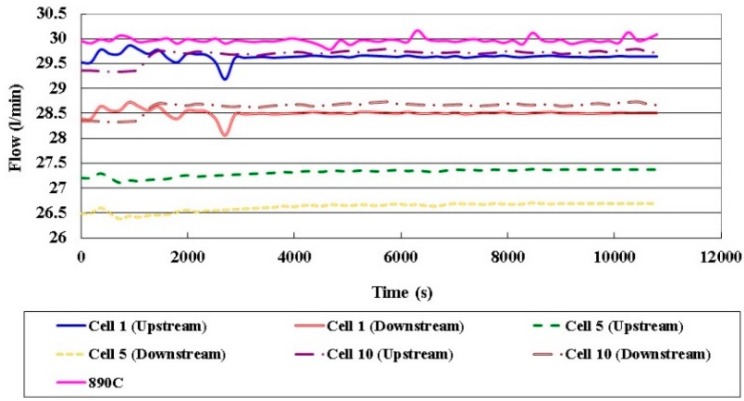
Comparison of local flow rates in different cells.

Figure 12 displays the local flow in different cells. The upstream flow rate exceeds the downstream flow rate, and the flow rates in Cell 1 and Cell 10 exceed those in Cell 5. Since the upstream flowing in Cell 1 and Cell 10 is close the air inlet, the gas is sufficient. The downstream flow rate is lower, because multiple snakelike runners (commonly called as “serpentine flow channels”) are used to improve the performance of the HT-PEMFC stack, and the internal distribution of the internal gas is nonuniform. 

### 3.4. Local Current Distribution in Different Cells 

**Figure 13:**
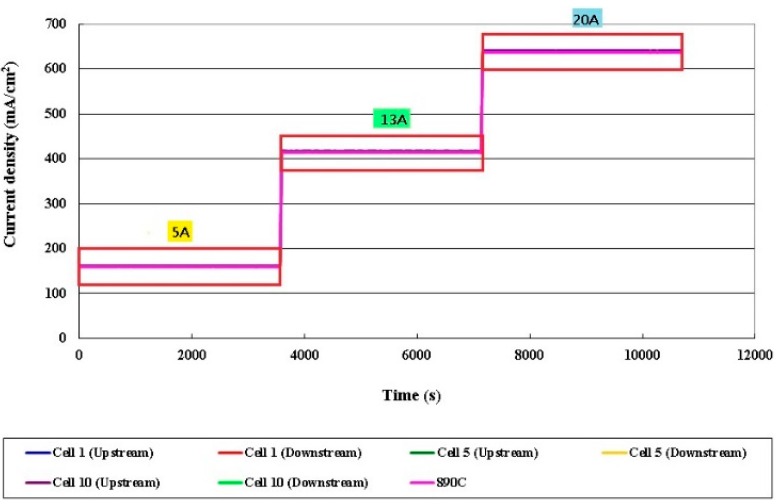
Comparison of local current densities in different cells.

**Figure 14:**
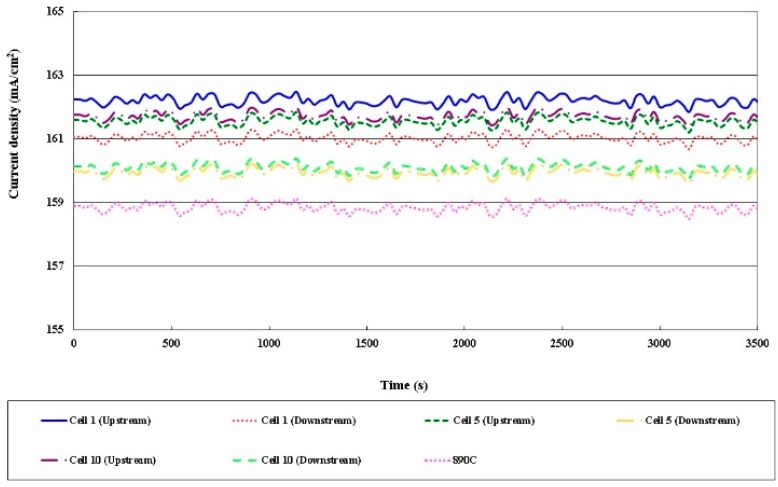
Comparison of local current densities in different cells (part of 5 A).

**Figure 15:**
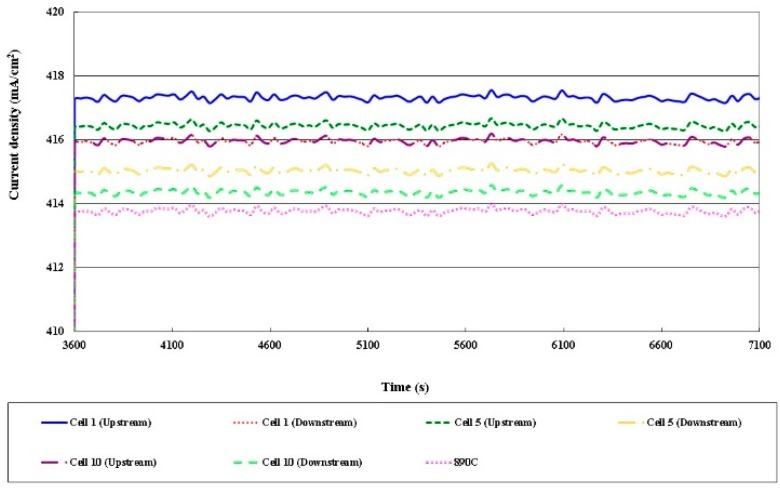
Comparison of local current densities in different cells (part of 13 A).

**Figure 16:**
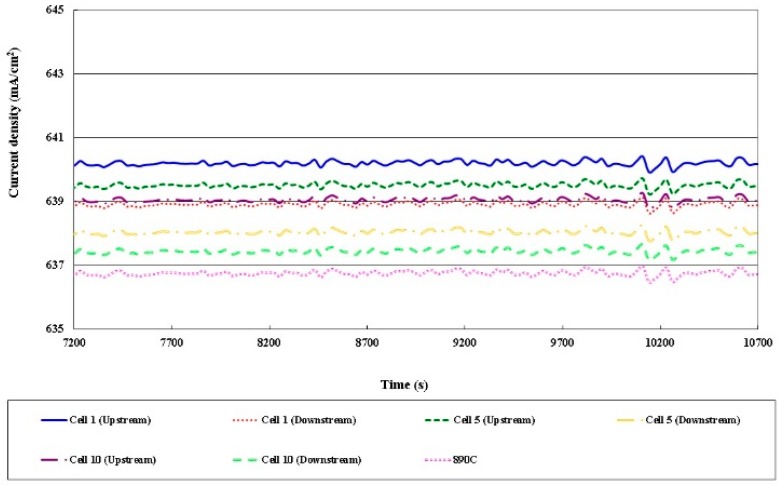
Comparison of local current densities in different cells (part of 20A).

Figure 13, Figure 14, Figure 15 and Figure 16 plot the local current density in different cells and this in-plane current does not directly represent the through-plane current flow through the catalyst layers and PEM (Proton Exchange Membrane). The current density in the HT-PEMFC stack varies with the current (5, 13, 20 A). The current density upstream exceeds that downstream, because the gas is present at the upstream inlet to initiate a electrochemical reaction, but the multiple serpentine flow channels cause the internal gas distribution to be nonuniform. The Nernst loss due to gas (especially oxygen) depletion increases toward the downstream part.

## 4. Conclusions

In this work, MEMS technology is utilized to develop a five-in-one micro-sensor on a 40-μm-thick stainless steel substrate. The protective layer is made of PI (Polyimide) with a favorable temperature tolerance. This five-in-one micro-sensor has five functions. It is characterized by its compactness, resistance to acid corrosion, favorable temperature tolerance, rapid response and usefulness in real-time measurement. 

The local temperature, flow and pressure information inside the HT-PEMFC stack are extracted successfully. The test result of the operating temperature of 160 °C and the constant current (5, 13, 20 A) shows nonuniform temperature and flow distributions in the HT-PEMFC stack.

The experiments indicated that the nonuniform temperature and flow distributions in the HT-PEMFC stack were responsible for a large internal temperature difference, nonuniform voltage and current distributions, and a hot stack. The pressure inside the HT-PEMFC stack was stable, but the air inlet upstream pressure was unstable at both the cathode and the anode.

## Figures and Tables

**Table 1:** Testing conditions for the HT-PEMFC stack.

Subject	Condition
Temperature of the stack (°C)	160
Cathode flow rate (Air) (slpm)	30
Anode flow rate (H_2_) (slpm)	5
Constant current (A)	5, 13, 20
Gas temperature	Room temperature
Reaction area (cm^2^)	31.4

**Table 2:** Specifications of the HT-MEA.

Subject	Specificationsis
HT-MEA	Advent energy
Membrane thickness	60~65 μm
Conductivity	8 × 10^−2^ S/cm
